# Determination of Biogenic Amines in Seawater Using Capillary Electrophoresis with Capacitively Coupled Contactless Conductivity Detection

**DOI:** 10.3390/molecules23051112

**Published:** 2018-05-08

**Authors:** Elbaleeq A. Gubartallah, Ahmad Makahleh, Joselito P. Quirino, Bahruddin Saad

**Affiliations:** 1 School of Chemical Sciences, Universiti Sains Malaysia, Penang 11800, Malaysia; 2 Chemistry Department, Faculty of Science, University of Khartoum, Khartoum 11115, Sudan; 3 Department of Chemistry, Faculty of Science, University of Jordan, Amman 11942, Jordan; Makahleh@hotmail.com; 4 Australian Centre for Research on Separation Science (ACROSS), School of Physical Sciences-Chemistry, University of Tasmania, Hobart 7001, Australia; joselito.quirino@utas.edu.au; 5 Fundamental & Applied Sciences Department and Institute for Sustainable Living, Universiti Teknologi PETRONAS, Seri Iskandar 32610, Perak, Malaysia

**Keywords:** capillary electrophoresis, capacitively coupled contactless conductivity detector, biogenic amines, seawater

## Abstract

A rapid and green analytical method based on capillary electrophoresis with capacitively coupled contactless conductivity detection (C^4^D) for the determination of eight environmental pollutants, the biogenic amines (putrescine, cadaverine, spermidine, spermine, tyramine, 2-phenylamine, histamine and tryptamine), is described. The separation was achieved under normal polarity mode at 24 °C and 25 kV with a hydrodynamic injection (50 mbar for 5 s) and using a bare fused-silica capillary (95 cm length × 50 µm i.d.) (detection length of 10.5 cm from the outlet end of the capillary). The optimized background electrolyte consisted of 400 mM malic acid. C^4^D parameters were set at a fixed amplitude (50 V) and frequency (600 kHz). Under the optimum conditions, the method exhibited good linearity over the range of 1.0–100 µg mL^−1^ (*R*^2^ ≥ 0.981). The limits of detection based on signal to noise (S/N) ratios of 3 and 10 were ≤0.029 µg mL^−1^. The method was used for the determination of seawater samples that were spiked with biogenic amines. Good recoveries (77–93%) were found.

## 1. Introduction

Biogenic amines (BAs) are basic organic compounds with aliphatic, aromatic, or heterocyclic structures. They are classified into mono or polyamines according to the number of amino groups they contain. In foods, BAs are formed by microbial decarboxylation processes of related amino acid [1,2,3]. In low concentrations, polyamines are essential for nucleic acid and protein synthesis [1]. Putrescine (PUT), cadaverine (CAD), spermidine (SPD), spermine (SPM), histamine (HIS), phenylethylamine (PEA), tyramine (TYR) and tryptamine (TRY) are considered to be the most important BAs that are found in foods [1,2]. BAs have also been proposed as indicators of food quality and freshness [2]. BAs can be found in different types of foods, such as fruits, vegetables, dairy products, meat, fish, beverages and fermented food [3]. The presence of BAs in significant concentrations can cause toxicity [3]. Table 1 shows the chemical properties of some of the important BAs. From the environmental point of view, it is interesting to monitor BAs in water bodies, as some BAs can react with nitrite to produce nitrosamines which are highly carcinogenic compounds [3]. Thus, the analysis of BAs in water samples has started to receive considerable interest lately [4,5].

Several analytical methods have been used for the determination of BAs. These methods mainly used gas chromatography (GC) [6,7], high performance liquid chromatography (HPLC) [8,9] and capillary electrophoresis (CE) [10,11]. Most of these techniques required chemical derivatization due to the lack of chromophores and to increase the sensitivity using ultraviolet (UV) and fluorescence detection. Recently there has been great interest in the use of direct detection to avoid the derivatization step. This is mainly due to the fact that (i) most derivatization reagents are expensive, (ii) a long derivatization time is required, (iii) side products associated with derivatization are frequently encountered, and (iv) the shelf-life of derivatization reagents are short [4,12]. CE has proven to be an interesting separation technique, mainly due to its superb resolving power in separating closely-related compounds (e.g., isomers, chirals) and the consumption of markedly reduced samples and reagents. Several papers have described the use of CE for the determination of BAs. As UV detection results in low sensitivities in CE, other detectors, such as amperometric [5], conductometric [13] and capacitively coupled contactless conductivity detections (C^4^D) [4,14], have been reported. Sensitivity improved by a factor of 100 when the C^4^D was used compared to the indirect UV detection for the determination of non-UV absorbing amines [15]. A further scrutiny of these papers also reveals that complicated background electrolytes (BGE) that contain cyclodextrin [15], crown ether [4,5,15] and α-hydroxyisobutyric acid [16] were used as the chiral selector.

In this work, we describe a simple and green CE–C^4^D method for the separation and quantitation of eight BAs (PUT, CAD, SPD, SPM, TYR, HIS, TRY and PEA). The chemical structures of the BAs are shown in Table 1. During the course of the method development, malic acid was used as the BGE component without the need for any other organic modifiers or chiral selectors, thus simplifying the procedure. The method was validated and qualified and applied for the determination of BAs in seawater.

## 2. Results

### 2.1. Chemicals and Reagents

All chemicals and solvents used were of analytical and chromatographic grade, respectively. Spermine tetrahydrochloride (SPM), spermidine trihydrochloride (SPD), cadaverine dihydrochloride (CAD), putrescine dihydrochloride (PUT), histamine dihydrochloride (HIS), tryptamine hydrochloride (TRP) and citric acid were purchased from Sigma–Aldrich (Steinheim, Germany). Tyramine hydrochloride (TYR) and propionic acid (99%) were from Fluka (Buchs, Switzerland). Formic acid (85%) was from QRёC, while acetic acid (99.85%) was from HmbG Chemicals. Tartaric (99.5%) and malic acids, methanol and acetonitrile (ACN) were obtained from Merck (Darmstadt, Germany). Succinic acid (99%) was from BDH Chemicals (Bridgeport, PA, USA). Hydrochloric acid was obtained from Lab Scan (Bangkok, Thailand) and trichloroacetic acid (TCA) was from R & M Chemicals (Essex, UK). Milli-Q water was produced from a Nanopure Diamond, Barnstead unit and was used throughout.

### 2.2. Preparation of Standard Solutions

Stock solution (1000 mg L^−1^) of a mixture of the eight BAs was prepared in water in a volumetric flask (10 mL). The solution was stored in the dark at 4 °C. Working solutions were prepared by appropriate dilution of the stock in water.

### 2.3. Seawater Samples

Seawater samples were collected on the 26–28 September 2016 from eight different places (Batu Ferringhi, Tanjung Bunga, Padang Kota, Bayan Lepas, Batu Maung, Teluk Kumbar, Tanjong Assam, Balik Pulau) around Penang Island, Malaysia. 

### 2.4. Preparation of Samples 

All samples were filtered through 0.22 µm nylon filter before introducing to the CE unit.

### 2.5. Instrumentation and Electrophoretic Conditions

Separations were performed on a 7100 capillary zone electrophoresis system (Agilent Technologies, Waldbronn, Germany) connected with C^4^D (eDAQ, Denistone East, Australia). The separations were obtained using a bare fused silica capillary with a capillary size of 95 cm × 50 µm i.d. (detection length, 10.5 cm from the outlet end of the capillary) supplied by Agilent Technologies (Waldbronn, Germany). Standards and samples were introduced hydrodynamically (50 mbar) for 5 s; other conditions are as shown in Table 2. Data acquisition was performed using licensed Power-Chrom software version 2.6.11 (eDAQ, Denistone East, Australia). The new capillary was activated by flushing for 15 min with 1.0 M NaOH, 15 min with 0.1 M NaOH and 20 min with water followed by 15 min with the BGE. Between injections, the capillary was preconditioned with 0.1 M NaOH, water and the BGE (each for 5 min). All standards, samples, BGE, and NaOH solutions were filtered through 0.2 µm nylon filter membranes (Agilent Technologies).

## 3. Results and Discussion

### 3.1. Capillary Electrophoresis Method Development

The initial electrophoretic conditions with C^4^D used were adopted from the work of Gong & Hauser [18] and Li et al. [4], who achieved the separation of the eight BAs using 150 mmol L^−1^, 18-crown-6 in 500 mmol L^−1^ acetic acid as the BGE. Thirty minutes of preconditioning and equilibrium time between every two injections was required. Under these conditions, the authors were able to separate the BAs in about 24 min. In order to shorten the run time and simplify the BGE, several parameters affecting the separation of BAs were studied.

#### 3.1.1. Selection of Background Electrolyte

Background electrolyte (BGE) is one of the most important parameters in CE method development. Several CE studies using different detectors have been developed for the determination of BAs. For C^4^D, it is important to keep the background conductivity as low as possible. This ensures that the small signals due to conductivity changes in the capillary between the excitation and pick-up electrode are amplified [19]. The pH of BGE solution used for the separation of BAs should be significantly different from the pK_a_ values of the BAs. Generally, the pH of the BGE should be 2 units larger or lower than the pK_a_ of the BAs, to ensure that the BAs are in the ionized form for optimum conductivity detection [20].

In this study, different types of weak organic acids were studied, including monocarboxylic acids (formic, acetic and propionic), dicarboxylic acids (oxalic and malonic), tricarboxylic acid (citric), unsaturated dicarboxylic acid (maleic) and hydroxyl dicarboxylic acids (malic and tartaric). These acids were selected based on their solubility in water and low conductivity to ensure high baseline stability [18]. Most of these acids succeeded in achieving the goal of ionization, but unluckily, did not result in satisfactory separation of all of the BAs. The use of acetic acid resulted not only in sensitive signals, but also, fast separations (~14 min). However, HIS and CAD was separated but TRY and TYR were unsolved. Promising separation was achieved using malic acid (Supplementary Figure S1). Therefore, malic acid was selected for further investigations.

#### 3.1.2. Effect of the pH and Concentration of Background Electrolyte

Besides the choice of organic acid as the BGE component, the selection of pH is of great importance in CE-C^4^D analysis as it can influence the mobility of analytes by modifying the electro-osmotic flow (EOF) velocity and the ionic charge of the analyte molecules [21]. The effect of pH on the separation of the BAs was tested over a pH range of 1.8–2.6, keeping other conditions constant (BGE: malic acid (300 mmol L^−1^); voltage (25 kV); injection time (5 s); capillary temperature, (24 °C); C^4^D conditions, frequency (600 kHz) and amplitude (100 V). The best result was obtained when operated at pH 2.0. (See Supplementary Figure S2). Either increasing the pH over 2.0 or decreasing it to 1.8 (mixing with another acid) resulted in very poor sensitivity.

The effect of malic acid concentration (100–500 mmol L^−1^) on the separation of BAs was also studied. The results showed that the resolution of the analytes was poor when 300 mmol L^−1^ was used. Good resolution with baseline separation was obtained when 400 mmol L^−1^ malic acid was used. When the concentration was increased to 500 mmol L^−1^, total overlap between SPM and SPD was observed. Using 350 and 450 mmol L^−1^, malic acid did not improve the separation. Therefore, 400 mmol L^−1^ malic acid was selected for further studies.

#### 3.1.3. Effect of Organic Modifiers

The effect of adding different organic modifiers (methanol, ethanol or ACN) to the BGE (5%, *v*/*v*) was studied. Both methanol and ethanol showed poor resolution for the BAs, while ACN resulted in acceptable resolution but with low sensitivity, in agreement with an earlier study [22]. Different percentages of ACN (1–10%) resulted in satisfactory resolution, but the sensitivity remained poor. Hence, no organic modifier was used for further optimization.

#### 3.1.4. Effect of Instrumental Parameters

##### Effect of Separation Voltage

In order to shorten the separation time and further improve the resolution, the effect of the applied voltage (20–30 kV) was studied. The best resolution with the shortest run time was obtained when 25 kV was applied. Joule heating is not applicable at this applied voltage because of the low conductivity of the BGE used.

##### Effect of Capillary Temperature

The capillary temperature affects the resolution in CE by affecting the viscosity of the BGE [23]. The effects of different temperatures (16–26 °C) were investigated. The best resolution with an acceptable run time was obtained when operated at 24 °C. Further increases in temperature deteriorated the resolution between SPD and CAD.

##### Optimization of C^4^D Parameters

In order to improve the sensitivity of the C^4^D detector, the frequency and amplitude were investigated. The frequency of C^4^D was studied from 300 to 1000 kHz. The highest peak area was obtained when 600 kHz was used. Furthermore, the amplitude was also studied from 20 V to 100 V. The highest signals were obtained when operated at 50 V.

**Figure 1:**
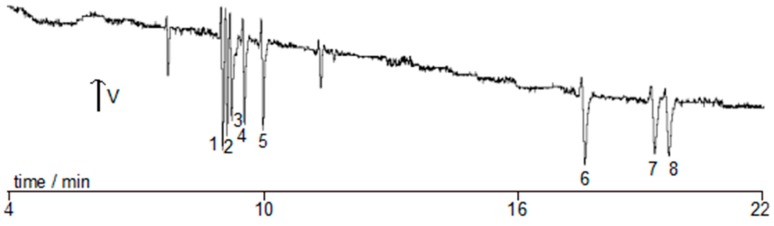
Typical electropherogram for the separation of eight biogenic amines under the optimum conditions mentioned in Table 2. Peak identity: SPM (1), SPD (2), HIS (3), CAD (4), PUT (5), PHE (6), TYR (7), and TRY (8).

The adopted conditions are summarized in Table 2, while Figure 1 shows a typical electropherogram of the standard BAs when separated under these conditions. The eight BAs were baseline separated within about 20 min, compared to the previous reported methods where 24 and 29 min were required [4,5].

### 3.2. Analytical Characteristics of the Method

The linearity of the method for all BAs was studied over wide range of concentrations (1.0–100.0 mg L^−1^ for PUT, SPD and SPM; 2.0–100.0 mg L^−1^ for PEA and TRY; 5.0–100.0 mg L^−1^ for HIS and TYR; 1.0–50.0 mg L^−1^ for CAD). The results are shown in Table 3. Good linearity, with correlation coefficients (*R*^2^) between 0.981 and 0.996, were obtained (*n* = 3). The limits of detection (LODs) for the analytes at signal-to-noise ratios of three and ten ranged between 0.016 and 0.029 mg L^−1^ (Table 3). The relative standard deviation (RSD) values for migration time were less than 6%.

The intra-day and inter-day precisions were tested using three different concentrations of standard mixture solutions. The intra-day precision was tested with six replicates in one day and the inter-day precision was tested by assays over six days. The intra-day and inter-day RSD were 5.8–9.1% and 4.0–9.7%, respectively. The recovery study was examined using seawater that was spiked with three different concentrations of BA mixture (10, 25 and 50 µg mL^−1^). It is indeed very encouraging to find that satisfactory recovery for all BAs was obtained (77–93%) (Table 4). It must also be pointed out that the seawater was only filtered before the CE-C^4^D analysis, and yet, there was no significant interference from the complex matrix. This is mainly due to the fact that conductivities of the background ions were suppressed when operated under the adopted conditions.

### 3.3. Analysis of Seawater Samples

The developed method was applied for the analysis of BAs in eight seawater samples collected around Penang Island. Before the analysis, these samples were filtered through 0.2 µm nylon membrane filter. BAs were not detected in the analyzed samples. The results obtained were in agreement with those reported for other environmental waters [4,5] where most BAs were not detected. At the moment, BAs are not subjected to any environmental regulations.

A comparison of LODs and recoveries of CE-C^4^D with other direct CE reported methods is summarized in Table 5. The results show that by using this method, the LODs obtained were lower when compared with previously reported methods using electrochemical and C^4^D detectors [4,21]. Also the LOD values were higher compared to HPLC combined with derivatization [8]. Meanwhile, the analysis time (~20 min) was shorter than the CE and HPLC methods reported by Li et al. (~24–29 min) [4,5] and Saaid et al. (~27 min) [8].

## 4. Conclusions

The simultaneous determination of eight BAs without derivatization using CE-C^4^D was demonstrated. These analytes were separated in about 20 min. Unlike the earlier CE-C^4^D work, our proposed method uses a very simple BGE (malic acid) and requires no sample pretreatment before the analysis. Earlier work used 18-crown-6 as the BGE component and SPE for the treatment of environmental water samples. The BAs were separated in about 25 min. The proposed CE-C^4^D thus offers an interesting alternative to replace the common method for BA analysis that involves the HPLC separation of derivatized analytes for UV or fluorescence detection, which requires significant amounts of environmentally unfriendly organic solvents. The proposed method offers remarkable selectivity, enabling the BAs to be analyzed in complex seawater samples without any pretreatment.

## Figures and Tables

**Table 1:** Some chemical properties of the studied biogenic amines (BAs) [13,17].

BA	Structure	pK Value
Putrescine (PUT)	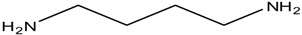	pK_1_ = 10.8; pK_2_ = 9.4
Cadaverine (CAD)	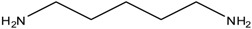	pK_1_ = 11.0; pK_2_ = 9.9
Spermidine (SPD)	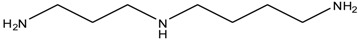	pK_1_ = 9.5; pK_2_ = 10.8; pK_3_ = 11.6
Spermine (SPM)	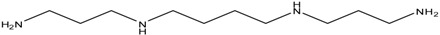	pK_1_ = 11.50; pK_2_ = 10.95; pK_3_ = 9.79; pK_4_ = 8.90
Histamine (HIS)	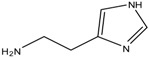	pK_1_ = 9.8; pK_2_ = 6.0
Tryptamine (TRY)	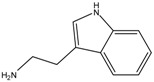	pK = 10.2
Tyramine (TYR)	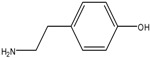	pK = 9.6
Phenylethylamine (PEA)	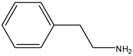	pK = 10.0

**Table 2:** Adopted capillary electrophoresis (CE)-capacitively coupled contactless conductivity detection (C^4^D) conditions.

Background electrolyte	400 mmol L^−1^ malic acid
Applied voltage	25 kV (normal polarity)
Capillary temperature	24 °C
Capillary	Bare fused silica (50 µm i.d. × 87 cm length)
Injection time	5 s
C^4^D parameters	Amplitude, 50 V; frequency, 600 kHz

**Table 3:** Analytical characteristics of the developed CE-C^4^D method.

BAs	Linear Range (mg L^−1^)	Regression Equation	*R* ^2^	LOD (µg L^−1^)
PUT	1.0–100	y = 0.375x + 3.715	0.988	27
CAD	1.0–50	y = 0.465x + 2.400	0.989	22
HIS	5.0–100	y = 0.367x + 6.082	0.981	28
SPD	1.0–100	y = 0.362x + 3.519	0.982	29
SPM	1.0–100	y = 0.430x + 3.725	0.991	24
PEA	2.0–100	y = 0.490x + 2.849	0.982	21
TYR	5.0–100	y = 0.626x + 0.104	0.985	16
TRY	2.0–100	y = 0.473x + 1.193	0.996	27

**Table 4:** Percent recoveries of BAs obtained from seawater that was spiked with different BA standards (*n* = 6).

BAs	Spiked Concentration, mg L^−1^		
	10	25	50
PUT	78.6	83.4	89.3
CAD	80.5	82.3	92.7
HIS	86.0	87.4	85.7
SPD	76.6	80.0	89.9
SPM	82.5	80.6	88.4
PEA	84.3	87.8	91.9
TYR	87.6	88.4	90.8
TRY	90.0	91.6	89.2

**Table 5:** Some capillary electrophoresis methods for the determination of BAs without derivatization.

Matrix	BAs	Detector	LOD	Recovery (%)	References
Rice spirit	TYR, TRY	Electrochemical	1.8 × 10^−7^ mol L^−1^2.3 × 10^−7^ mol L^−1^	102	[24]
Tuna fish	PUT, CAD, SPD, HIS	Conductivity	0.15–50 mg kg^−1^	92–102	[25]
Drosophila brains	TYR	Cyclic Voltammetry	2.5 nM	-	[26]
Beer	TYR	Electrochemical	4.3 mg L^−1^	-	[22]
BeerWine	PUT, CAD, HIS, SPD, SPM, PEA, TYR, TRY	MS/MS	1–2 µg L^−1^	87–113	[27]
Water	PUT, CAD, HIS, SPD, SPM, PEA, TYR, TRY	C^4^D	44.3–149 µg L^−1^	86.9–104	[4]
Milk	PUT, CAD, SPD, SPM	Amperometry	10^−7^–4 × 10^−7^ M	-	[28]
Water	CAD, HIS, SPD, SPM, PEA, TYR, TRY	Amperometry	10.1–42.6 µg L^−1^	71.6–101	[5]
BeerWineSalamiCheese	PUT, CAD, HIS, TRY, TYR	Conductometry	2–5 µmol L^−1^	86–103	[13]
BeerWine	TRY, TYR	Amperometry	5.8 × 10^−7^ M15.0 × 10^−7^ M	97.596	[29]
Fermented dairy products	CAD, HIS, SPD, TYR, PUT	C^4^D	41–98 µg L^−1^	89–103	[16]
Sea water	PUT, CAD, HIS, SPD, SPM, PEA, TYR, TRY	C^4^D	16–29 µg L^−1^	77–93	This study

## Supplementary Materials

The following are available online at http://www.mdpi.com/1420-3049/23/5/1112/s1, Figure S1: Effect of different organic acids as BGE on the separation of the BA, Figure S2: Effect of pH of BGE on the separation of BAs.
