# Supplementary material for: Determination of Biogenic Amines in Seawater Using Capillary Electrophoresis with Capacitively Coupled Contactless Conductivity Detection
Source: Molecules. 2018 May 8;23(5):1112. doi: 10.3390/molecules23051112 (PMC6100306; doi:10.3390/molecules23051112)
Supplement: Supplementary file 1 [file molecules-23-01112-s001.pdf]

Supplementary Material:

Determination of Biogenic Amines in Seawater using Capillary Electrophoresis with Capacitively Coupled Contactless Conductivity Detection

Elbaleeq A. Gubartallah [1,2,*], Ahmad Makahleh [3], Joselito P. Quirino [4] and Bahruddin Saad [1,5,*]

1 School of Chemical Sciences, Universiti Sains Malaysia, Penang 11800, Malaysia
2 Chemistry Department, Faculty of Science, University of Khartoum, Khartoum 11115, Sudan
3 Department of Chemistry, Faculty of Science, University of Jordan, Amman 11942, Jordan; Makahleh@hotmail.com
4 Australian Centre for Research on Separation Science (ACROSS), School of Physical Sciences-Chemistry, University of Tasmania, Hobart 7001, Australia; joselito.quirino@utas.edu.au
5 Fundamental & Applied Sciences Department and Institute for Sustainable Living, Universiti Teknologi PETRONAS, Seri Iskandar 32610, Perak, Malaysia
* Correspondence: baleeqoz@hotmail.com (E.A.G.); bahruddin.saad@utp.edu.my (B.S.); Tel.: +605-368-7683; Fax: +605-365-5905

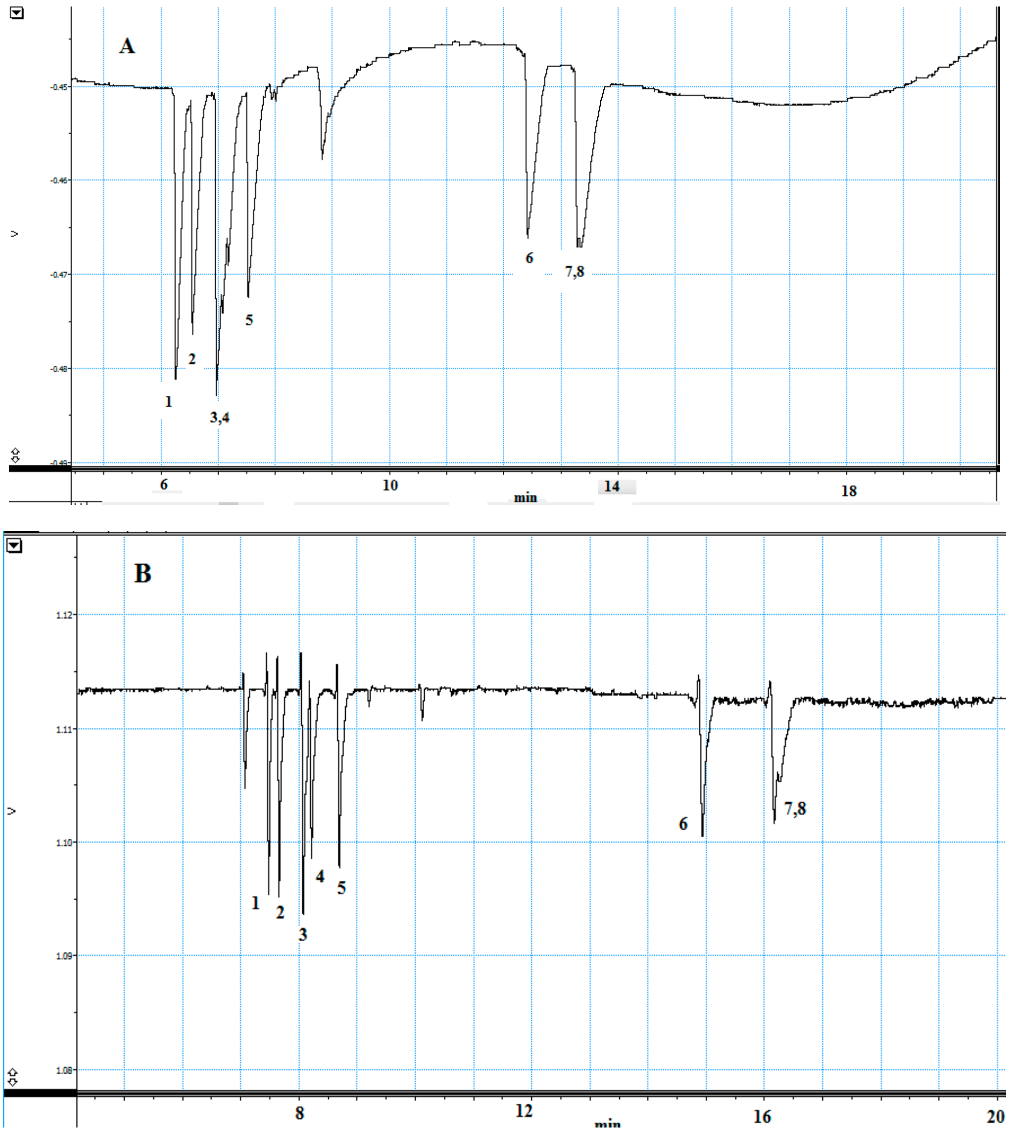

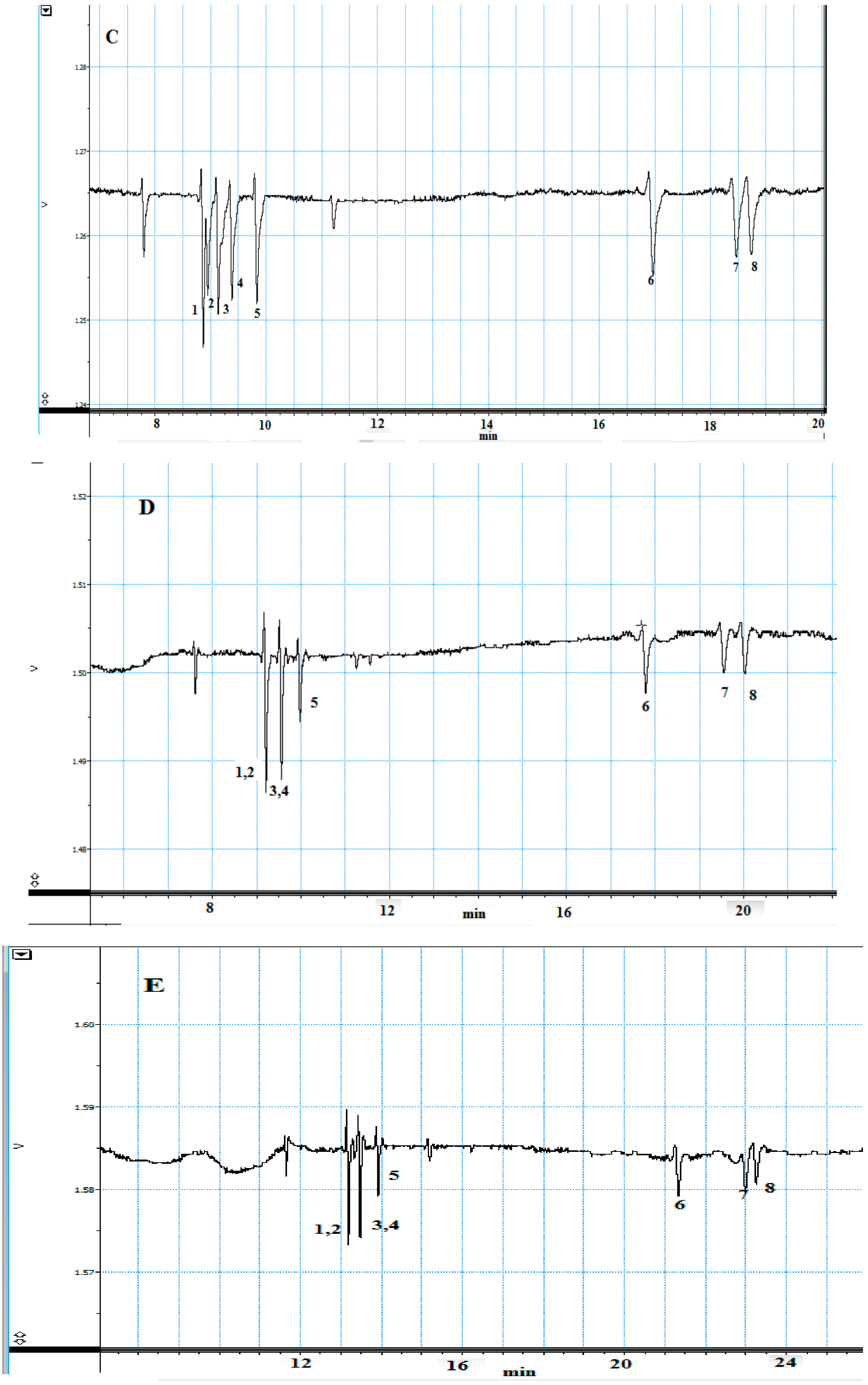
C
1.28
1.27
1.26
1.25
1.24
1
2
3
4
5
6
7
8
8
10
12
14
min
16
18
20
D
1.52
1.51
1.50
1.49
1.48
1,2
3,4
5
6
7
8
8
12
min
16
20
E
1.60
1.59
1.58
1.57
1,2
3,4
5
6
7
8
12
16
min
20
24

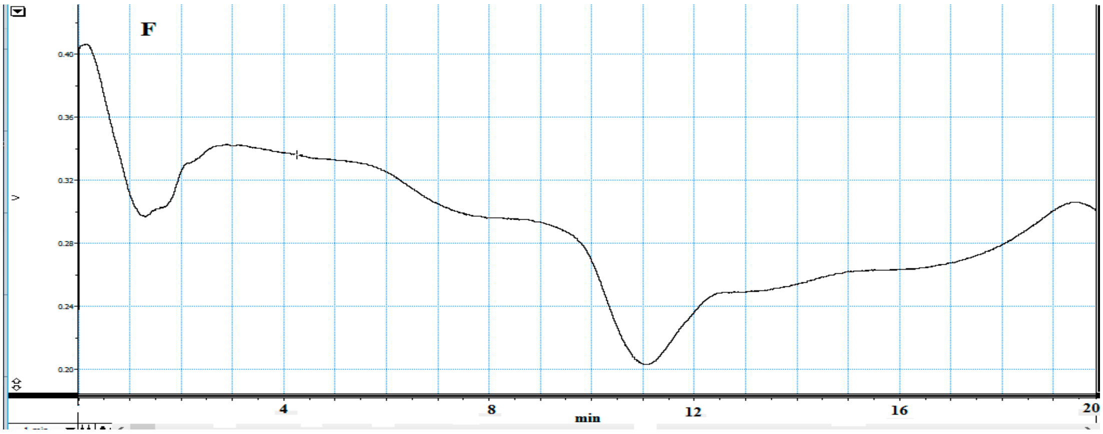

Supplementary Figure S1. Effect of different organic acids as BGE on the separation of the BA. (A) acetic acid, (B) formic acid, (C) malic acid, (D) citric acid, (E) tartaric acid and (F) trifluoroacetic acid. Peak identity: SPM (1), SPD (2), HIS (3), CAD (4), PUT (5), PHE (6), TYR (7), and TRY (8). CE conditions: acid (300 mmol L^{-1}); voltage (25 kV); injection time (5 s); capillary temperature, (24^{0}C); C^4D conditions, frequency (600 kHz) and amplitude (100 V).

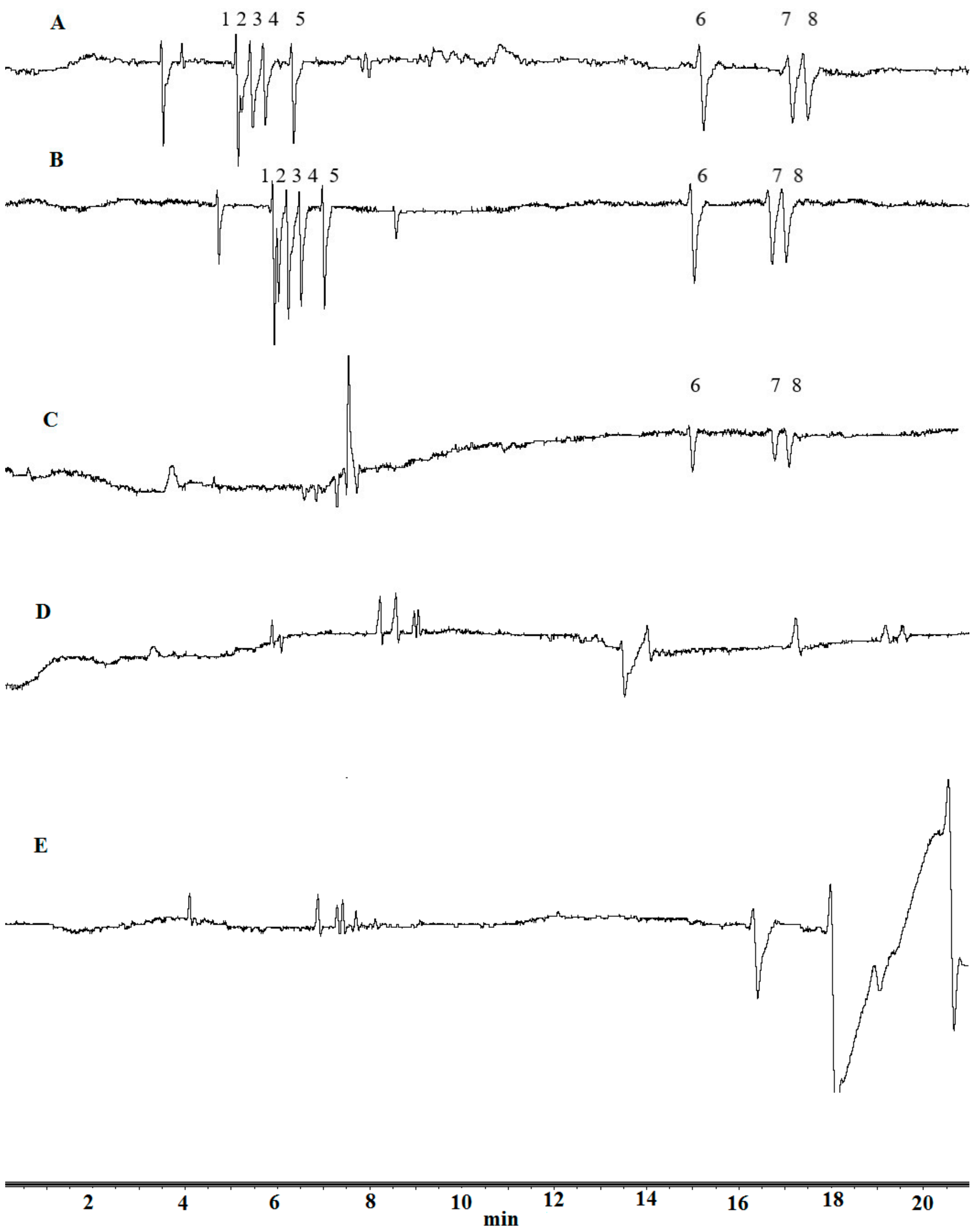

Supplementary Figure S2. Effect of pH of BGE on the separation of BAs. (A) 1.8, (B) 2.0 (C) 2.2 (D) 2.4 and (E) 2.6. CE conditions: malic acid (300 mmol L^{-1}); voltage (25 kV); injection time (5 s); capillary temperature, (24^{0}C); C^4D conditions, frequency (600 kHz) and amplitude (100 V).
